# Long-Term Evaluation of Pseudoexfoliation Syndrome Post-Cataract Extraction

**DOI:** 10.3390/jpm13050818

**Published:** 2023-05-12

**Authors:** Karny Shouchane-Blum, Alon Zahavi, Noa Geffen, Yoav Nahum, Eitan Livny, Irit Rosenblatt, Ruti Sella, Irit Bahar, Amir Sternfeld, Dan Gaton

**Affiliations:** 1 Ophthalmology Department and Laboratory of Eye Research Felsenstein Medical Research Center, Rabin Medical Center, Petach Tikva 4941492, Israel; karny.blum@mail.huji.ac.il (K.S.-B.); noatal1122@gmail.com (N.G.); yoav.nahum@gmail.com (Y.N.); eitanlivny@gmail.com (E.L.); iritbahar@gmail.com (I.B.); shtery@gmail.com (A.S.); gaton@tauex.tau.ac.il (D.G.); 2 Sackler Faculty of Medicine, Tel Aviv University, Tel Aviv 6997801, Israel

**Keywords:** cataract, pseudoexfoliation syndrome, glaucoma, pseudophakia

## Abstract

The study aimed to examine the effect of cataract extraction on ophthalmologists’ ability to detect pseudoexfoliation (PXF) syndrome. A total of 31 patients admitted for elective cataract surgery were enrolled in this prospective comparative study. Prior to surgery, patients underwent slit-lamp examination and gonioscopy conducted by experienced glaucoma specialists. Subsequently, patients were re-examined by a different glaucoma specialist and comprehensive ophthalmologists. Pre-operatively, 12 patients were diagnosed with PXF on the basis of a Sampaolesi line (100%), anterior capsular deposits (83%), and pupillary ruff deposits (50%). The remaining 19 patients acted as controls. All patients were re-examined 10–46 months post-operatively. Of the 12 patients with PXF, 10 (83%) were correctly diagnosed post-operatively by glaucoma specialists and 8 (66%) by comprehensive ophthalmologists. There was no statistically significant difference in PXF diagnosis. However, detection of anterior capsular deposits (*p* = 0.02), Sampaolesi lines (*p* = 0.04), and pupillary ruff deposits (*p* = 0.01) were significantly lower post-operatively. Diagnosis of PXF is challenging in pseudophakic patients as the anterior capsule is removed during cataract extraction. Therefore, PXF diagnosis in pseudophakic patients relies mainly on the presence of deposits at other anatomical sites, and careful attention to these signs is required. Glaucoma specialists may be more likely than comprehensive ophthalmologists to detect PXF in pseudophakic patients.

## 1. Introduction

Pseudoexfoliation (PXF) syndrome is an age-related, systemic disorder with unique ocular manifestations that is estimated to affect 60–70 million people worldwide [1]. In the eye, PXF is characterized by the presence of deposits on various anterior segment structures, conjunctiva, anterior capsule, pupillary margin, corneal endothelium, iridocorneal angle, and the trabecular meshwork [2]. A Sampaolesi line, which is a formation of brownish pigmented line anteriorly to Schwalbe’s line in the iridocorneal angle, is a common characteristic of PXF [3]. However, the diagnosis of PXF is often based on the anterior capsule, which is the main site where PXF is noted [4].

PXF is considered the leading cause of secondary glaucoma, as it is the cause of pseudoexfoliation glaucoma [5,6]. PXF glaucoma is an aggressive form of glaucoma with faster progression and higher fluctuating intraocular pressures that are harder to control pharmacologically compared with primary open-angle glaucom; it often requires surgical intervention [3,7,8]. Its diagnosis is, therefore, paramount for optimal lifelong patient care.

On routine cataract extraction, most of the anterior capsule, which is the main ocular site of PXF, is removed during capsulorhexsis. Consequently, the diagnosis of PXF after cataract extraction may become challenging. In our previous short-term study, we showed that the ability to diagnose PXF is significantly decreased one week after cataract surgery [9]. However, PXF is a chronic progressive disease, and signs of PXF in general tend to become more evident with the passage of time. Therefore, a longer post-operative follow-up period may improve diagnostic abilities. The goal of this current prospective study is to evaluate the long-term success in diagnosing PXF- and PXF-related anatomical features in pseudophakic patients at least 10 months post-surgery.

## 2. Materials and Methods

This was a prospective comparative study design conducted at a single tertiary medical center. The study was approved by the institutional ethics committee and complies with the tenets of the Declaration of Helsinki. Patients over 18 years of age admitted for elective cataract extraction were invited to participate, and all participants signed informed consent forms prior to enrolment. Prior to surgery, each participant underwent a thorough slit-lamp examination, including gonioscopy (Zeiss 4 Mirror Gonioscopy Lens, Carl Zeiss AG, Oberkochen, Germany), for detection of PXF. The examination was performed by one of five experienced glaucoma specialists before and after pupillary dilation; its results were then used to assign patients to the “PXF” (study) and “Non-PXF” (control) groups. Only the operated eye was examined in order to avoid diagnosis bias due to possible PXF signs present in the other eye. The physician documented the presence or absence of PXF deposits in the following sites: anterior capsule, pupillary ruff, iridocorneal angle, and corneal endothelium. The presence or absence of a Sampaolesi line was also documented. Each patient was re-examined (including gonioscopy) and documented similarly by a different experienced glaucoma specialist and comprehensive ophthalmologists at least 10 months after cataract extraction. Participants were invited to the re-examination in a random order, and all specialists were masked to the pre-operative examination, i.e., they did not know if the patient was in the study or control group, and to each other’s evaluation. Two glaucoma specialists and three comprehensive ophthalmologists took part in the post-operative examination.

Exclusion criteria included subjects diagnosed with closed-angle glaucoma, secondary glaucoma other than PXF, any ocular condition preventing a reliable examination of the anterior segment, and any intraoperative complication. Subjects who underwent previous ocular surgery either prior to cataract extraction or between the first and second study examinations were also excluded from the study.

Statistical analysis was performed, which used a T-test for continuous parameters and Fisher’s exact test for categorical parameters. McNemar’s test was used for the analysis of paired dichotomic parameters. A *p* value of <0.05 was considered significant.

## 3. Results

A total of 31 patients were enrolled in the study. The control and study groups were similar in demographic and baseline characteristics, including age, gender, side of the operated eye, personal and family history of glaucoma, cup to disc ratio, and best corrected visual acuity. None of the study or control group patients had post-operative complications, and none had undergone another intraocular surgery during the study’s follow-up time. The study group consisted of pre-operatively diagnosed PXF patients (*n* = 12), while the control group included non-PXF patients (*n* = 19). The time of re-examination ranged from 10 to 46 months (average 23 months). Prior to surgery, the diagnosis of PXF was based on the following features: a Sampaolesi line (100%), anterior capsule deposits (83%), and pupillary ruff deposits (50%). In the pre-operative examination, there was no detection of PXF deposits on the corneal endothelium. The pre-operative examination conducted by a glaucoma specialist was compared independently to the post-operative examination conducted by another glaucoma specialist, as well as to the post-operative examination conducted by a comprehensive ophthalmologist.

**Figure 1:**
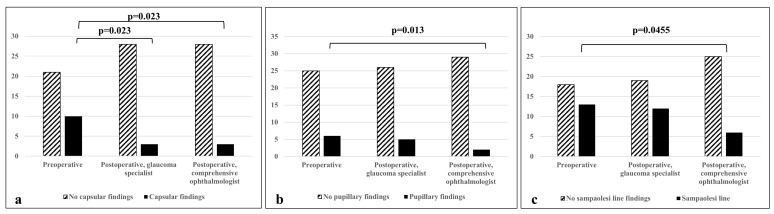
Difference in detecting various PXF findings between non-glaucoma and glaucoma specialists. (**a**) Detection of deposits on anterior capsule; (**b**) detection of deposits on pupillary ruff; (**c**) detection of a Sampaolesi line. *p* < 0.05 is considered statistically significant.

**Figure 2:**
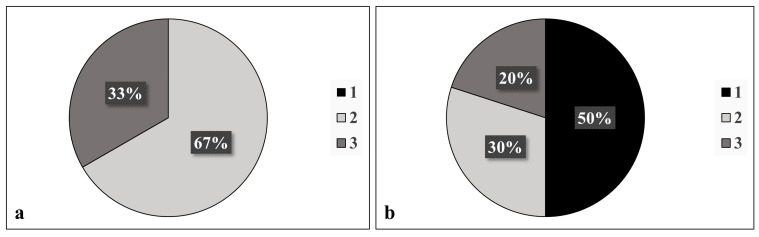
Number of sites involved (anterior capsule, pupillary ruff, iridocorneal angle, corneal endothelium, or presence or absence of a Sampaolesi line) in diagnosis of pseudoexfoliation (**a**) before and (**b**) after surgery, as was documented by a glaucoma specialist.

When compared with a glaucoma specialist, there was no statistically significant difference in the diagnosis of PXF before and after cataract extraction (*p* = 0.48); 10/12 patients were correctly diagnosed with PXF post-surgery based on the following features: a Sampaolesi line (80%); pupillary ruff deposits (50%); anterior capsule deposits (33.3%); and exfoliation deposits, other than a Sampaolesi line, in the iridocorneal angle (10%). After surgery, there was a significant decrease in the detection of anterior capsule deposits (*p* = 0.02, Figure 1, Table 1). Before surgery, 67% of PXF patients were found to have two sites involved in the disease, while 33% had three sites involved. No diagnosis was based on the involvement of only one site prior to surgery. However, after surgery, 50% of patients diagnosed with PXF were found to have only one site involved (Figure 2).

When compared with the post-operative examination of the comprehensive ophthalmologist, there was no statistically significant difference in the diagnosis of PXF before and after cataract extraction (*p* = 0.13). However, it may be clinically significant that 4/12 (33%) patients were misdiagnosed after surgery by the comprehensive ophthalmologist. Moreover, there was a significant decrease in the detection of anterior capsule deposits (*p* = 0.02), and, unlike the comparison to the glaucoma specialist, a decrease was also noted in the detection of pupillary ruff deposits (*p* = 0.01) and a Sampaolesi line (*p* = 0.04) by the comprehensive ophthalmologist (Figure 1, Table 1).

## 4. Discussion

This study investigated the potential difficulties of diagnosing PXF in pseudophakic patients. It was found that when most of the anterior capsule is removed (main site of PXF fibrillar protein accumulation), the importance of meticulous examination of the pseudophakic eye for PXF signs in other ocular sites increases in order to minimize the risk for under diagnosis of PXF, which can lead to less follow-up than advisable for PXF patients. The use of gonioscopy and specific attention for pupillary ruff deposits and a Sampaolesi line was shown to be crucial for proper diagnosis. Previous studies showed that ophthalmologists’ adherence to gonioscopy, even in known glaucoma patients, is quite low and ranges between 46 and 85% [10,11,12,13]. The Glaucoma Adherence and Persistency Study was published in 2007 and is one of the most extensive studies performed to evaluate compliance of open-angle glaucoma patients to topical therapy, as well as ophthalmologists’ adherence to the preferred practice patterns. In this study, the charts of 300 open-angle glaucoma patients were reviewed. It was found that only 50% of patients had undergone a gonioscopy examination [13]. Interestingly, the same results were found in the “Glaucoma Care and Conformance with Preferred Practice Patterns” study performed back in 1996 [11], which showed 51% adherence to gonioscopy. Many reasons were raised to explain the low rate of gonioscopy examination: patients had undergone gonioscopy conducted by a previous ophthalmologist, while gonoscopy is a hard-to-perform examination and requires experience, as well as being time-consuming. For example, central corneal thickness measurement, which is a parameter that became part of the routine examination of glaucoma patients much later than gonioscopy, has similar or even higher adherence rates, probably because it is automated and a much easier test to perform [13]. It can be safely assumed that gonioscopy is performed at even lower rates in non-glaucoma patients and by non-glaucoma specialists. In our study, in which all examiners were asked to use gonioscopy during their clinical evaluation, only 6/13 patients were correctly diagnosed as having a Sampaolesi line when examined by a comprehensive ophthalmologist, as opposed to 12/13 patients when examined by a glaucoma specialist. Other signs that may raise suspicion of PXF during clinical examination in pseudophakic eyes including a poorly dilated pupil, pseudophakodonesis, and an atrophic pupillary margin.

PXF was discovered by Finnish ophthalmologist John G. Lindberg in 1917 [3]. Its pathophysiology was previously studied in diverse populations of different geographic regions, ethnicities, and races. Older age as well as Scandinavian or Mediterranean descent are implicated as PXF risk factors [4,5]. The reported rates of PXF vary greatly, ranging from 0% in Eskimos to 40.6% in patients aged over 80 years old in Iceland [3]. Genetic studies implicated lysyl oxidase-like protein 1 (LOXL1) gene mutations in elastin metabolism abnormalities, resulting in the characteristic fibrillary deposits noted in PXF patients [1,2,3]. Electron microscopy studies showed PXF fibrils to be amyloid-like filamentous material with amorphous ground substance [5]. The PXF material is produced intracellularly in the non-pigmented ciliary epithelial cells and trabecular endothelial cells, and then released extracellularly [14]. It is deposited in the anterior segment structures, including the pupillary margin, zonules, and anterior lens capsule. Additionally, pseudoexfoliative material was identified in blood vessel walls as well as the lung, myocardium, kidney, liver, gall bladder, and meninges [5]. The deposits are associated with the development of PXF-related comorbidities.

The importance of PXF diagnosis is well established, and it has been associated with several systemic conditions [15,16,17,18]. In the Blue Mountains Eye Study, pseudoexfoliation was associated with a history of angina, hypertension, acute myocardial infarction, and stroke after multivariate adjustment [19]. In a study examining the association of PXF and systemic vascular disease in a South Indian population, 930 patients with PXF were compared with 476 non-PXF patients scheduled for cataract surgery [20]. In the study, PXF patients had statistically significant higher systolic blood pressure levels and ECG abnormalities than non-PXF controls. Several studies also associated PXF with abdominal aortic aneurysms. In a prospective study evaluating 55 patients with abdominal aortic aneurysms compared to 41 controls with carotid artery occlusion, a significantly higher proportion of the study group had pseudoexfoliation as compared to the controls [21]. Additionally, in the study, histopathological examinations of aortic wall samples obtained from pseudoexfoliation patients showed pseudoexfoliation material in the vessel wall not seen in normal age-matched control samples. In a different study conducted by Djordjevic-Jocic et al., 60 patients with pseudoexfoliation syndrome, 60 with pseudoexfoliation glaucoma, 60 with primary open-angle glaucoma, and 60 with cataract (control group) were examined for the presence of abdominal aortic aneurysms [22]. The frequency of abdominal aortic aneurysms in patients with pseudoexfoliation syndrome or pseudoexfoliation glaucoma was 14.8%, significantly higher than that of other groups. Systemic vascular pathology’s association with PXF is thought to include genetic LOXL1 allele variability and environmental factors [3]. The pseudoexfoliative material deposition in vessel walls may contribute to increased vascular resistance and blood flow changes, as clinically noted in these studies. Additional systemic PXF-related manifestations include peripheral vascular disease, neurosensory hearing loss, renal artery stenosis, and Alzheimer’s-like dementia [18,19,20,21,22].

Ocular conditions associated with PXF include zonular laxity and possible crystalline lens dislocation as a potential outcome of fibrillar material deposition on the zonules, increased rates of nuclear cataracts, and PXF glaucoma [3,23]. Higher rates of nuclear cataracts in PXF eyes are hypothesized to be a result of aqueous humor changes and possible relative ocular ischemia, leading to lens metabolism alterations, resulting, in turn, in cataract formation [3,18]. PXF-related zonular laxity combined with poor pupillary dilation also increases the risk of complicated cataract extraction with vitreous loss, often requiring additional surgical techniques for successful outcomes [1,18,24,25]. PXF-related zonular changes increase the risk of zonular rupture and lens dislocation by 3-to-10-fold, and vitreous loss by 5-fold [18]. Histological studies showed proteolytic damage to the zonular fibers due to pseudoexfoliative material deposition, with resulting zonular laxity [1]. Pre-operative evaluation of zonular laxity can be challenging. Clinical signs include iridodonesis and phacodonesis, up to lens subluxation. The usefulness of ultrasound biomicroscopy for the evaluation of zonular stability is controversial [1,23]. A small pupil and poor pharmacological mydriasis occurs due to pseudoexfoliative material deposition in the iris tissues with associated atrophy and relative iris muscle hypoxia due to vascular abnormalities, as demonstrated in angiographic studies [25]. Poor mydriasis limits capsulorhexis size, thereby increasing surgical manipulations for cataract extraction, zonular strain, and the risk of capsular tear and vitreous loss [1]. Low corneal endothelial cell counts are also associated with PXF, which poses some PXF eyes at increased risk for corneal decompensation following phacoemulsification [1,18]. The increased risk of cataract surgery in PXF eyes has led experts in recent years to perform cataract extraction early in the disease to avoid the increased risk of poor mydriasis, zonular instability, and hard nuclear cataracts [18].

Post-surgical complications in PXF eyes include intraocular lens instability [7,26]. An extensive meta-analysis by Vazquez-Ferreiro et al. concluded that patients with PXF are more susceptible to intraocular lens dislocation, necessitating surgical treatment [17]. In a study by Davis et al., 86 intraocular lenses explanted within the capsular bag were analyzed via light microscopy [26]. The main condition associated with late lens dislocation was pseudoexfoliation, accounting for 50% of cases, while the mean time for explantation was 8.4 years in pseudoexfoliation patients. Capsular fibrosis and capsular contraction were significant findings. Zonular insufficiency was the cause of all within-the-bag dislocations. The zonular weakness was associated with pseudoexfoliation, possible intraoperative zonular tension, and tractional forces exerted via post-operative capsular fibrosis and capsulorhexis phimosis. The rate of post-surgical lens instability can be decreased by avoiding intraoperative zonular stress, performing a larger capsulorhexis, implanting capsular tension rings when zonular laxity is noted, and considering implantation of a 3-piece intraocular lens in PXF patients. Post-surgical intraocular pressure spikes are more common in PXF eyes and warrant specific attention, particularly in PXF glaucoma patients. Additionally, PXF is associated with a damaged aqueous blood barrier, resulting in increased baseline anterior chamber inflammatory response [25]. Due to the disrupted aqueous blood barrier, the post-surgical inflammatory reaction is higher in PXF eyes, which may warrant additional anti-inflammatory treatment [7].

Increased risk of secondary glaucoma was extensively reported in relation to PXF [18,27], which was described in up to 50% of eyes with PXF. PXF is considered the leading cause of secondary glaucoma, which is often characterized by an aggressive course relative to primary open-angle glaucoma that often necessitates surgical intervention [4,5]. As a result, PXF glaucoma patients have a poorer prognosis compared to primary open-angle glaucoma patients, and legal blindness is more common due to PXF glaucoma compared to primary open-angle glaucoma [6,8]. Without treatment, PXF glaucoma progresses three times more rapidly than primary open-angle glaucoma [7]. At diagnosis, PXF glaucoma patients have a higher frequency of optic nerve damage compared to primary open-angle glaucoma patients, with associated visual field damage [4]. Elevated intraocular pressure in PXF eyes is assumed to be a result of increased outflow resistance in the trabecular meshwork due to pigment and pseudoexfoliative material deposition [3]. Higher intraocular pressure in PXF eyes was reported regardless of glaucoma compared to non-PXF eyes, and the rate of progression to glaucoma in increased intraocular pressure eyes is higher in PXF than non-PXF eyes [4,5,6]. Intraocular pressure fluctuations are more common in PXF glaucoma patients, and is associated with a more severe clinical course [7]. Additional factors other than increased intraocular pressure are thought to contribute to the glaucomatous damage seen in PXF patients. Vascular abnormalities leading to decreased ocular and retrobulbar perfusion may contribute to the optic nerve damage [3,4,5]. Abnormal lamina cribrosa elastic tissue constituents decrease its resistance to mechanical deformation and may contribute to the progressive nature of the disease [3,4,5,7]. This outcome may explain why eyes with PXF are at a higher risk of developing glaucoma compared to eyes without PXF at the same intraocular pressures [4,5]. Environmental factors that increase oxidative stress are also suspected of contributing to the progression of PXF to glaucoma. Anterior chamber levels of increased oxidative stress markers were documented, with corresponding low levels of antioxidants [7]. These markers may also be responsible in part for the increased rates of cataract and zonular instability noted in PXF patients. To date, treatment for PXF glaucoma relies on different modalities for intraocular pressure reduction, including pharmacological, laser, and surgical procedures. Currently, no treatment is available for other possible contributing factors for disease progression, such as vascular changes, tissue laxity, and oxidative stress factors.

During cataract extraction, a large portion of the anterior capsule is removed, along with characteristic PXF deposits when present. The typical anterior capsule appearance of PXF-affected lenses consists of three zones: a central disc, a clear intermediate zone, and a peripheral granular zone [3]. The intermediate clear zone is hypothesized to result from iris rubbing on the anterior capsule, removing the pseudoexfoliative fibrillar material. When examining a pseudophakic patient, ophthalmologists are often faced with a diagnostic dilemma as one of the main clinical signs of the condition is absent, and specific attention is, therefore, warranted to secondary sites of PXF fibrillar material accumulation. This aspect was addressed in previous studies, including the Blue Mountains Eye Study [5]. In the study that included 4433 patients, PXF diagnosis estimates were corrected for patients before and after cataract extraction due to the difficulties in diagnosis of PXF in cases where a portion of the anterior capsule with the characteristic PXF fibrillar deposits is missing. This problem led to patients with bilateral aphakia or pseudophakia being excluded from the study. Of the patients in the study diagnosed with PXF, 52% had PXF unilaterally, with similar prevalence in the right and left eye. Bilateral involvement increased with age from 0% under age 60 to 75% in patients aged 80 years old or older. Other studies show that the chances of developing PXF in a fellow uninvolved eye range from 6.8% to 40% depending on age [25]. Additionally, conjunctival biopsy in a clinically unaffected eye is often positive [25], suggesting that PXF functions in a bilateral condition, even if it is often significantly asymmetrical.

Secondary sites of PXF fibrillar material accumulation include the corneal endothelium, pupillary ruff, iridocorneal angle, and the formation of a Sampaolesi line [3]. Iris pigment epithelium atrophy and dispersion can result in peripupillary iris atrophy and transillumination defects. These are different in location and appearance from the spoke wheel configuration slit-like iris transillumination defects seen in pigmentary glaucoma. Increased trabecular meshwork pigmentation is also seen in PXF and is often uneven in distribution, unlike in pigmentary glaucoma patients. The characteristic Sampaolesi line seen in PXF often aids in diagnosis, yet is not pathognomonic and can also be found in pigment dispersion and chronic inflammation [25]. Corneal endothelium pigmentation is also noticed in PXF-affected eyes. The pigmentary changes may precede the classic deposition of PXF fibrillary material on the anterior capsule of the lens [6]. During long-term follow-up after cataract extraction, new PXF deposition may be seen on the anterior surface of the intraocular lens or the anterior vitreous face [28]. All these sites should be carefully examined during patient evaluation.

In investigating the long-term PXF diagnostic challenges in pseudophakic patients 10 to 46 months following cataract extraction, a trend was found for PXF diagnosis difference before and after surgery, albeit without proven statistical significance. However, it may be clinically significant as 2/12 patients were misdiagnosed after surgery. As there are various sites in which PXF may present, its diagnosis can rely on the involvement of one or more sites. Pre-operatively, 2–3 sites were identified as having PXF signs, while post-operatively, 50% were diagnosed with PXF based on a single site. A Sampaolesi line and pupillary ruff deposits were identified as the most probable sites for post-operative PXF signs identification. Unexpectedly, pupillary ruff margin deposit rates were relatively low in our study despite the special attention given to this anatomical landmark. Additionally, no PXF deposits were noted on the artificial intraocular lenses in this long-term study. Although uncommon, deposits on the anterior surface of artificial lenses were reported and should be searched for in suspected cases [28,29,30]. PXF deposition in such cases is opposite to that seen on the anterior capsule of the crystalline lens, with the center clear of deposits, thus allowing good visual acuity [30].

The study’s limitations include a relatively small study cohort, which may limit the statistical analysis. Additionally, PXF may form deposits on the anterior surface of the artificial lens over time due to fibrillar protein deposition similar to that seen on the crystalline lens [28,29,30]. This outcome was not seen in our study. A longer follow-up period may show such deposits. Another limitation is that examiners were aware that they were examining study patients. This knowledge can result in a higher than usual suspicion level for PXF signs. In order to minimize this bias and create some level of blinding to the examiners, control patients were included in the blinded study.

## 5. Conclusions

Although not statistically significant, the findings of this long-term study strengthen those of the previous short-term study [9], indicating that PXF identification can be challenging in pseudophakic eyes, and emphasizing the importance of a thorough pre-operative evaluation of a cataract extraction candidate and proper documentation of the pre-operative clinical findings for future follow-up. The ophthalmologist should document deposits on the anterior capsule, pupillary ruff, iridocorneal angle, and corneal endothelium, as well as the presence or absence of Sampaolesi line. Documentation of deposits of the anterior capsule are the most critical pre-operatively, since most will be removed during capsulorhexis at the time of surgery and will be difficult or impossible to detect post-operatively. This finding is particularly important in cases of glaucoma patients, in which the course of the disease may be more aggressive when PXF is the causative factor. This study has also shown the importance of gonioscopy before, but mostly after, surgery, which may be underperformed, as the sites of PXF deposit seen in gonioscopy become more significant after cataract surgery.

## Figures and Tables

**Table 1:** Comparison of pseudoexfoliation (PXF) findings between pre- and post-operative examination.

Finding		Pre-Operative	Post-Operative			
			Glaucoma Specialist	*p* 1	Comprehensive Ophthalmologist	*p* 2
Deposits on anterior capsule	Y	10 (32.3%)	3 (10%)	0.023 *	3 (10%)	0.023 *
	N	21 (67.7%)	28 (90%)		28 (90%)	
Deposits on pupillary border	Y	6 (19.4%)	5 (16.1%)	1.00	2 (6%)	0.013 *
	N	25 (80.6%)	26 (83.9%)		29 (94%)	
Sampaolesi line	Y	13 (41.9%)	12 (38.7%)	1.00	6 (19.4%)	0.0455 *
	N	18 (58.1%)	19 (61.3%)		25 (80.6%)	

* *p* < 0.05 is considered significant. *p* 1 compares pre-operative with post-operative examination by a glaucoma specialist. *p* 2 compares pre-operative examination with post-operative examination by a comprehensive ophthalmologist. PXF pseudoexfoliation, Y yes, N no.

## Data Availability

All data generated or analyzed during this study are included in this published article (data transparency).
